# The Dichotomous Nature of AZ5104 (an EGFR Inhibitor) Towards RORγ and RORγT

**DOI:** 10.3390/ijms20225780

**Published:** 2019-11-17

**Authors:** Kaja Karaś, Anna Sałkowska, Iwona Karwaciak, Aurelia Walczak-Drzewiecka, Jarosław Dastych, Rafał A. Bachorz, Marcin Ratajewski

**Affiliations:** 1 Laboratory of Epigenetics, Institute of Medical Biology, Polish Academy of Sciences, Lodowa 106, 93-232 Lodz, Poland; kaja.karas@gmail.com (K.K.);; 2 Laboratory of Transcriptional Regulation, Institute of Medical Biology, Polish Academy of Sciences, Lodowa 106, 93-232 Lodz, Poland; isachrajda@cbm.pan.pl; 3 Laboratory of Cellular Immunology, Institute of Medical Biology, Polish Academy of Sciences, Lodowa 106, 93-232 Lodz, Poland; adrzewiecka@cbm.pan.pl (A.W.-D.); jdastych@cbm.pan.pl (J.D.); 4 Laboratory of Molecular Modeling, Institute of Medical Biology, Polish Academy of Sciences, Lodowa 106, 93-232 Lodz, Poland; rafal@bachorz.eu

**Keywords:** RORgamma (nuclear receptor ROR-gamma isoform 1), RORgammaT (nuclear receptor ROR-gamma isoform 1), RORC (RAR related orphan receptor C), Th17 (T helper 17 cell), AZ5104, EGFR (epidermal growth factor receptor)

## Abstract

The *RORC* (RAR related orphan receptor C) gene produces two isoforms by alternative promoter usage: RORγ (nuclear receptor ROR-gamma isoform 1) and RORγT (nuclear receptor ROR-gamma isoform 1). Both proteins have distinct tissue distributions and are involved in several physiological processes, including glucose/lipid metabolism and the development of Th17 lymphocytes. Previously, we developed a stably transfected reporter cell line and used it to screen a library of kinase inhibitors. We found that AZ5104 acts as an RORγ agonist at low micromolar concentrations. Molecular docking analysis showed that this compound occupies the ligand binding domain of the receptor with a significant docking score. However, analysis of the biological activity of this compound in Th17 cells revealed that it downregulates RORγT expression and Th17-related cytokine production via inhibition of SRC-ERK-STAT3 (SRC proto-oncogene - extracellular regulated MAP kinase - signal transducer and activator of transcription 3). We thus identified a compound acting as an agonist of RORγ that, due to the inhibition of downstream elements of EGFR (epidermal growth factor receptor) signaling, exerts different biological activity towards a Th17-specific isoform. Additionally, our results may be relevant in the future for the design of treatments targeting signaling pathways that inhibit Th17-related inflammation in certain autoimmune disorders.

## 1. Introduction

The discovery of Th17 cells resulted in a revision of the paradigm that T-helper lymphocytes divide into two antagonistic Th1 and Th2 subpopulations that regulate two distinct modes of immune response. Th17 lymphocytes secrete IL-17A and IL-17F that, together with IL-21, IL-22, and GM-CSF2 (colony stimulating factor 2 (granulocyte-macrophage), create a Th17-specific set of cytokines that determine the biological function of these cells. These lymphocytes play crucial roles in host defense against numerous pathogens, including *Bacillus anthracis* [1], *Staphylococcus aureus* [2], and *Candida albicans* [3]. Indeed, the lack of Th17 cells observed in the immunological deficiency IgE syndrome (Job’s syndrome) causes recurring pneumonia and chronic mucocutaneous candidiasis [4,5]. However, in contrast to the physiological host defense function, Th17 cells also have negative functions associated with their role in the pathogenesis of certain autoimmune disorders, such as rheumatoid arthritis [6], psoriasis [7], multiple sclerosis [8], ankylosing spondylitis [9], and Crohn’s disease [10]. The differentiation of Th17 cells is controlled by many transcription factors, including STAT3 (signal transducer and activator of transcription 3) [11,12,13,14,15], IRF4 (interferon regulatory factor 4) [16], BATF (basic leucine zipper ATF-like transcription factor) [17,18], and RORγT (nuclear receptor ROR-gamma isoform 2) [19]. Of these factors, only RORγT exhibits Th17-specific expression and is therefore considered the master regulator of Th17 cell differentiation, similar to t-bet in Th1 cells [20], GATA3 (GATA binding protein 3) in Th2 cells [21], and FOXP3 (forkhead box protein P3) in Tregs [22].

RORγT is one of two protein products of the *RORC* (RAR related orphan receptor C) gene (NR1F3, nuclear receptor subfamily 1 group F member 3), and the other product, RORγ, is 21 amino acids longer. These receptors belong to the nuclear receptor subfamily of retinoic acid receptor-related orphan receptors (RORs), which also includes RORα (NR1F1) and RORβ (NR1F2). All these proteins are transcription factors that modulate gene activation by binding coactivators in a ligand-dependent manner [23,24]. RORγ/RORγT have the typical domain structure characteristic of other nuclear receptors [25], consisting of an *N*-terminal A/B domain, a DNA-binding domain (DBD/C domain), a hinge region (domain D) and a *C*-terminal ligand binding domain (LBD/E domain). The RORγ/RORγT isoforms are generated by transcription of *RORC* from two different promoters [26,27,28]. As mentioned earlier, RORγT is exclusively expressed in Th17 cells, where it orchestrates the expression of several cytokines [19,29]. In contrast, RORγ is broadly expressed [30] and regulates the circadian rhythm and lipid/glucose metabolism [31,32,33].

Due to the involvement of RORγ/RORγT in the development of the immune system, the regulation of metabolism, and the pathogenesis of autoimmune diseases and increasing evidence for their involvement in cancer biology, they have become the putative targets for drug design [34]. Indeed, an increasing number of articles have reported new compounds that modulate the activity of these receptors in agonistic [35] and inverse agonistic manners [36], with potential for use in adoptive cell therapy (ACT) or the treatment of select autoimmune diseases.

In this work, we present the identification of the compound AZ5104 as a specific agonist for the RORγ isoform in HepG2 cells. We also report that this compound negatively influenced the expression of the RORγT isoform and, as a consequence, inhibited the expression of RORγT-dependent interleukins and the differentiation of Th17 lymphocytes. Thus, we provide a new promising compound for potential therapeutic applications to treat autoimmune diseases with the Th17 component. In addition, the results of our study may also help to explain the molecular mechanism of the more frequent *S. aureus* infections in cancer patients treated with EGFR (epidermal growth factor receptor) inhibitors.

## 2. Results

### 2.1. Identification of AZ5104 as a RORγ Activator

**Figure 1:**
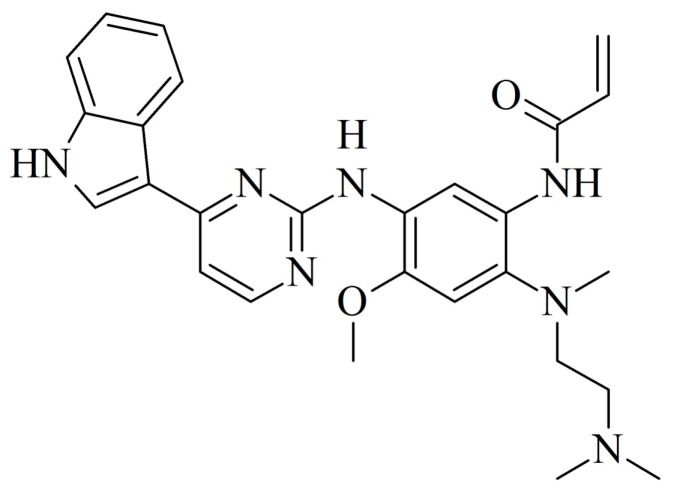
Structure of AZ5104, which was identified as an activator of RORγ (nuclear receptor ROR-gamma isoform 1) by screening the L1600 kinase inhibitor library (TargetMol).

**Figure 2:**
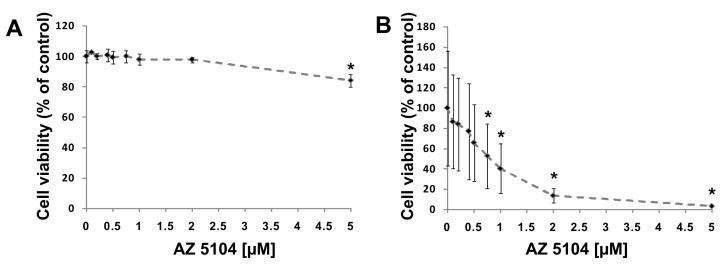
Individual cytotoxic effects of AZ5104. (**A**) Results of the neutral red uptake assay in HepG2 cells treated with increasing concentrations of AZ5104 for 24 h. Mean ± SD, *n* = 6. (**B**) Results of cell viability determined with the CellTiter-Glo assay in CD4+ cells differentiating towards Th17 cells in the presence of AZ5104 for 5 days. Mean ± SD, *n* = 8. * *p* < 0.05 compared with control cells.

**Figure 3:**
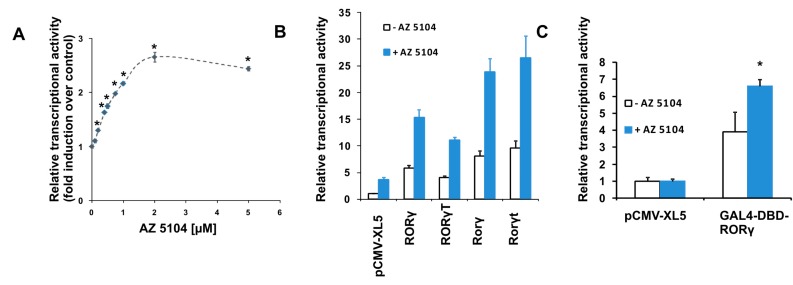
Individual biological effects of AZ5104. (**A**) AZ5104 induces RORγ-dependent transcription in the RORγ-HepG2 reporter cell line. Mean ± SD, *n* = 6. (**B**) AZ5104 potentiates the activity of human RORγ and RORγT and mouse Rorγ and Rorγt overexpressed in the RORγ-HepG2 reporter cell line. Mean ± SD, *n* = 6. Luciferase activity was standardized per corresponding secreted alkaline phosphatase activity, which was used as transfection efficiency control. Results were normalized to the activity of nontreated, pCMV-XL5-transfected cells. (**C**) AZ5104 induces the activity of the GAL4-DBD RORγ fusion protein in the HepG2-pGL4.35 reporter cell line. Mean ± SD, *n* = 6. * *p* < 0.05 compared with control treatment. Luciferase activity was standardized per corresponding secreted alkaline phosphatase activity, which was used as transfection efficiency control. Results were normalized to the activity of nontreated, pCMV-XL5-transfected cells.

To search for new substances that modulate RORγ activity, we screened the L1600 Kinase Inhibitor Library (TargetMol) with a previously validated [37] RORγ-HepG2 reporter cell line. We identified 13 compounds (Figure S1 and Table S1) in the initial chemical library screening, and these compounds were further processed in a two-step analysis that included: determination of dose-response curves and the ability to induce expression of the RORγ-dependent *G6PC* gene [38,39]. Only AZ5104 (Figure 1), an irreversible EGFR inhibitor [40], passed validation (true positive) while other twelve compounds were considered as false positives, (the false positive rate (FPR) was 2% which is significantly lower than observed by the other authors [41]). We then analyzed AZ5104 cytotoxicity in HepG2 cells, and the analysis showed that this compound is well tolerated by these cells at concentrations as high as 5 µM (Figure 2A), while in Th17 cells, exerted much higher toxicity (Figure 2B and Figure S2). The dose–effect curve indicated that 2 µM AZ5104 was optimal for the transactivation of RORγ in the reporter cells (Figure 3A). Next, we examined how overexpression of human RORγ and RORγT and mouse Rorγ and Rorγt in the RORγ-HepG2 reporter cell line influence the response to AZ5104. As shown in Figure 3B, we observed synergistic effects of protein overexpression and the examined compound. Interestingly we did not observe similar effects of this compound towards RORα and RORβ nuclear receptors (Figure S3). To show that the effects of the AZ5104 are at least in part mediated by the interactions of this compound with the ligand binding domain of RORγ, we used another reporter system in which the LBD domain of the RORγ receptor was fused to the GAL4 DNA-binding domain [42]. As expected, we observed a statistically significant induction of the reporter after treatment with AZ5104 (Figure 3C).

**Figure 4:**
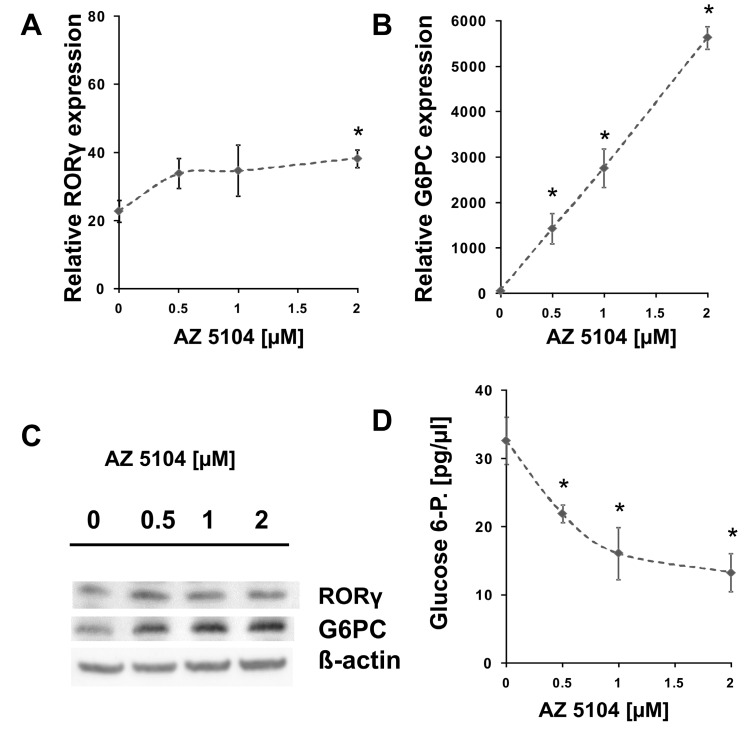
Effect of AZ5104 on the mRNA expression of *RORγ* (**A**) and *G6PC* (**B**) in HepG2 cells. HepG2 cells were treated with increasing concentrations of AZ5104 for 24 h and then collected for RNA extraction. The mRNA expression of *RORγ* and *G6PC* was determined by real-time RT-PCR and normalized to that of the housekeeping genes *HPRT1*, *HMBS,* and *RPL13A*. Mean ± SD, *n* = 3. * *p* < 0.05 compared with control cells. (**C**) Effect of AZ5104 on the RORγ and G6PC proteins in HepG2 cells as determined using Western blotting. (**D**) Effect of AZ5104 on the glucose 6-phosphate levels in HepG2 cells. Mean ± SD, *n* = 3. * *p* < 0.05 compared with control cells.

HepG2 cells treated with AZ5104 showed a small (1.6-fold) but statistically significant induction of RORγ mRNA (Figure 4A); however, we did not observe similar results on protein level (Figure 4C). Notably, in the samples treated with increasing concentrations of AZ5104, we observed a substantial dose-dependent increase in the mRNA (Figure 4B) and protein (Figure 4C) expression of *G6PC,* a gene known to be regulated by RORγ [38,39]. This was accompanied by the decrease in levels of glucose 6-phosphate (Figure 4D), which is a substrate for G6Pase. It should be noted that AZ5104 has the ability to induce *G6PC* expression much more (20- to 50-fold induction depending on the experiment) than is apparent from its RORγ transactivatory function. This may suggest that AZ5104 also influences a signal transduction pathway leading to the induction of another RORγ-independent factor regulating *G6PC* expression, though the results from the use of siRNA methodology (Figure S4) showed that the contribution of RORγ in the process is significant.

### 2.2. Molecular Docking Analysis

**Figure 5:**
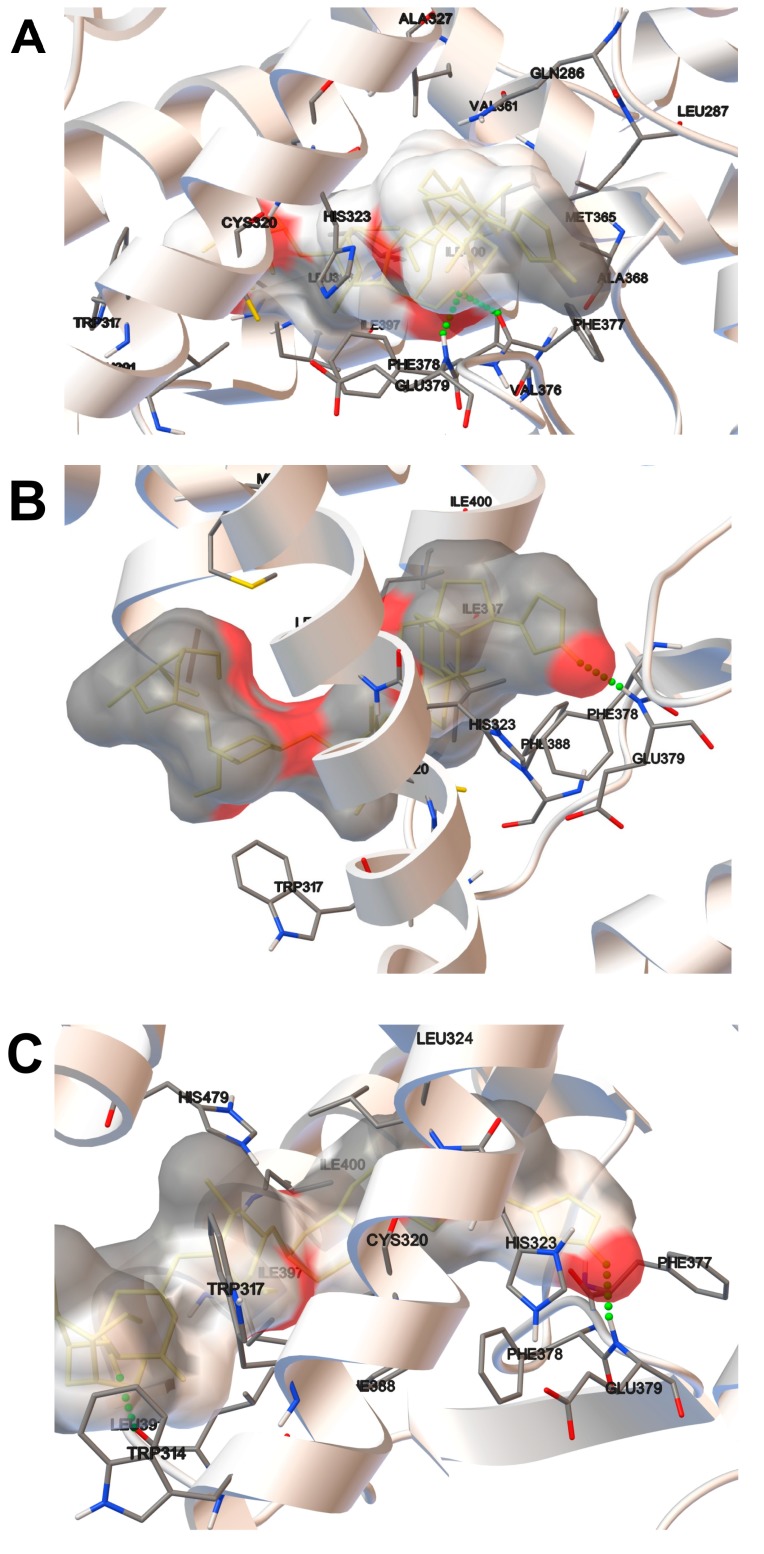
Molecular docking analysis of AZ5104 binding to the LBD (ligand binding domain) of RORγ: 3L0J (**A**), 3B0W A (**B**), and 3B0W B (**C**). Hydrogen bonds are shown as green dots.

In the current study, we considered 3 host domains of RORγ in the context of binding to AZ5104 within the molecular docking approach. The estimated free energies of binding are presented in Table 1, and all final structures are presented in Figure 5.

The host domain is represented as a white ribbon, and only the residues in close contact with the ligand are explicitly shown. The interactions with these explicitly named residues contribute to the overall binding. Due to the complicated nature of intermolecular interactions, it is not possible to precisely decompose interactions into each contribution from separate residues. However, we can assume that the origin of some fraction of the interaction energy is provided by the hydrogen bonds. Among the three considered ligand–host complexes, two (i.e., AZ5104 bound to the 3L0J and 3B0W_B domains) constitute explicit hydrogen bonds (Figure 5A,B). For the 3B0W_B (Figure 5C) system, such explicit hydrogen bonding was not observed. In the case of all considered domains, the compound generally tends to occupy the same binding pocket of the host. In the case of 3L0J and 3B0W_B, the ligand adopts a similar orientation where the indole part of the ligand is located deeper in the binding pocket. In the case of the 3B0W_A domain, a different orientation is preferred, but due to lower interaction energy in an overall picture, this orientation seems to be slightly less favorable. The 3B0W domain forms an explicit hydrogen bond with the AZ5104 molecule. Here, the amino group of the glutamic acid residue (GLU379) interacts with a carbonyl group of the ligand. In the case of 3L0J, the second domain forms hydrogen bonds with the ligand, and AZ5104 occupies almost the same space, but the hydrogen bond is between the carbonyl group of the phenylalanine residue (PHE377) and the amine group of AZ5104. The magnitude of an interaction of all the considered domains with the guest system is similar. Additionally, a slightly weaker interaction was observed for 3B0W_A and 3B0W_B compared to that for 3L0J. The molecular docking results described shortly above do not point out clearly the most favorable domain. From the point of view of the current study, all of them are equally likely to occur in the experiment. Only the further investigation carried out at the level of theory incorporating the quantum effects might provide additional insight and thus the final resolution.

### 2.3. AZ5104 Inhibits Th17-Specific Genes Expression

**Figure 6:**
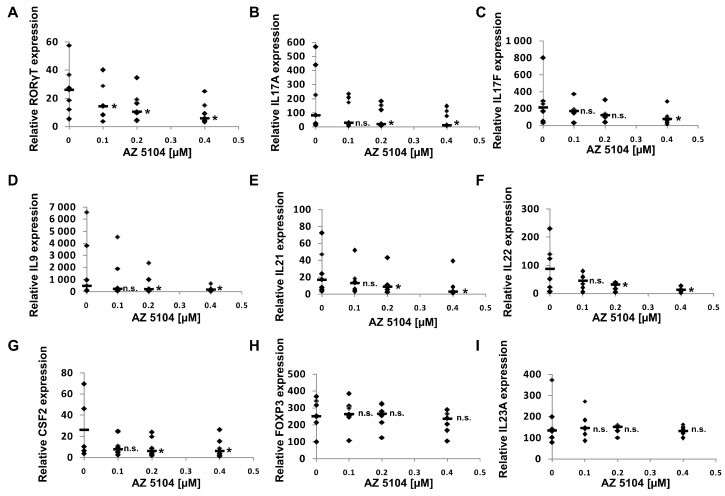
Effect of AZ5104 on the expression of selected genes in human Th17 cells. Naive human CD4+ cells were treated with increasing concentrations of AZ5104 and cultured under Th17 polarizing conditions for 5 days. Then, the cells were collected for RNA extraction. The expression of the *RORγT* (**A**), *IL17A* (**B**), *IL17F* (**C**), *IL9* (**D**), *IL21* (**E**), *IL22* (**F**), *CSF2* (**G**), *FOXP3* (**H**) and *IL23A* (**I**) genes was determined by real-time RT-PCR. The results were normalized to the housekeeping genes *HPRT1*, *HMBS,* and *RPL13A*. The data are presented as statistical dot plots with the median value (bars) of seven independent cultures originating from seven different donors (*n* = 7), * *p* < 0.05 compared with control; n.s.—not significant.

**Figure 7:**
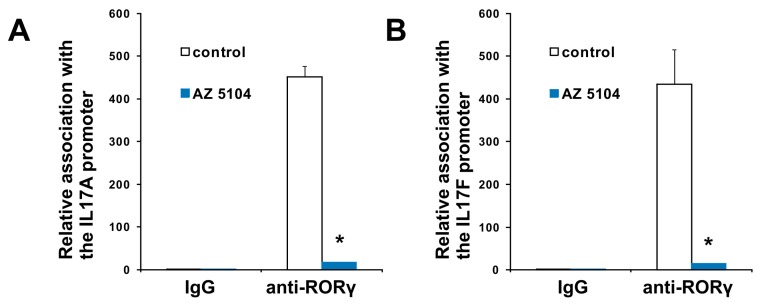
Results of chromatin immunoprecipitation showing that AZ5104 lowers RORγT occupancy at the *IL17A* (**A**) and *IL17F* (**B**) promoters. Data are presented as mean ± SD, *n* = 3 (from three different donors), * *p* < 0.05 compared with control cells.

**Figure 8:**
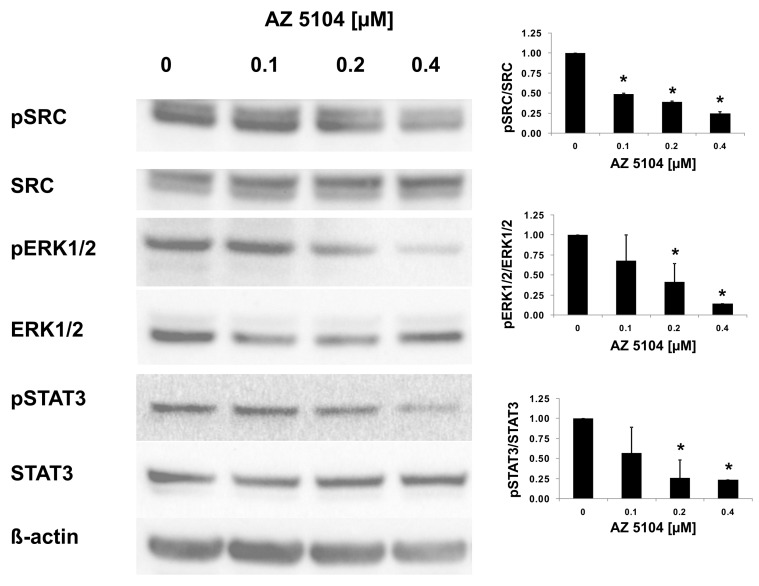
AZ5104 inhibits the phosphorylation of kinases downstream of EGFR. Human CD4+ cells differentiating towards Th17 lymphocytes were treated with increasing concentrations of AZ5104 for 1 h and then lysed, and pSRC, pERK1/2, and pSTAT3 levels were analyzed using Western blotting (left panel). Results of densitometric analysis were performed using ImageJ (right panel). Data are presented as mean ± SD, *n* = 3 (from three different donors), * *p* < 0.05 compared with control cells.

In the next series of experiments, we used human Th17 cells that exclusively express the shorter isoform of the *RORC* gene, RORγT. Both isoforms share the majority of the amino acid sequence, differing by only 21-amino acids in the *N*-terminal A/B domain. Their ligand binding domains are identical, which is why we expected that AZ5104 would activate RORγT-dependent transcription in Th17 cells as it did in HepG2 cells. However, when we analyzed the cytotoxicity of the compound in CD4+ cells differentiating into Th17 lymphocytes, we noticed that concentrations above 500 nM significantly decreased cell viability (Figure 2B). Unexpectedly, analysis of *RORγT* expression using real-time RT-PCR revealed that AZ5104 decreased these expression levels in a dose-dependent manner. This decrease was associated with decreases in *IL17A/IL17F* expression, the expression of other Th17-related cytokines (Figure 6), e.g., *IL9* [43], *IL21* [15], *IL22* [44] and *CSF2* [45], and IL-17 secretion (Figure S5). Such results were not observed for the *FOXP3* and *IL23A* [46] genes (Figure 6H,I). Further analysis indicated that AZ5104 decreased RORγT occupancy at the *IL17A/F* promoters (Figure 7). In another approach, we used fully differentiated Th17 to check whether shorter exposure of cells to the AZ5104 could have different effects. Analyzing cell viability, we observed that concentrations above 0.5 µM are also cytotoxic (above 20%) to these cells (Figure S2), and expression of the selected Th17 genes is already decreased (Figure S6). One explanation for these results is that in Th17 cells, AZ5104 is not able to act as an agonist of RORγT because the observed cytotoxicity prevents the compound from reaching an effective intracellular concentration. Alternatively, some putative compound-mediated signaling events do not allow AZ5104 agonist activity. In that respect, the dose-dependent inhibitory effects of AZ5104 on the expression of *IL17A/IL17F* and other Th17 cytokines are consistent with the hypothesis that this compound affects the signaling pathway regulating tissue-specific *RORγT* expression. Because AZ5104 was previously identified as an EGFR inhibitor [40], we decided to analyze the phosphorylation status of downstream elements of this signaling pathway. We observed a strong inhibition of phosphorylation levels of pSRC, pERK1/2, and pSTAT3 in CD4+ cells treated with AZ5104 while differentiating towards Th17 cells (Figure 8). Interestingly, AZ5104 had no effect on the SRC-ERK1/2-STAT3 pathway in the HepG2 cell line and, noteworthy, phosphorylation of STAT3 on Tyr705 was not visible in these cells (Figure S7) which suggests tissue-specific differences in the signaling network [47].

## 3. Discussion

In the human genome, 48 genes encoding proteins belonging to the nuclear receptor family have been identified thus far [48]. These genes express a significantly higher number of proteins due to differential promoter usage and alternative splicing, creating isoforms with different tissue distributions and functions [26,49,50]. Nuclear receptors play important roles in development and homeostasis [51,52] and are molecular targets for various therapies, including anti-cancer therapies, e.g., tamoxifen targeting ERα [53], enzalutamide targeting AR [54], hormone replacement, e.g., benzothiophene raloxifene targeting ER [55], immune system modulation dexamethasone targeting GR [56] and metabolic diseases, e.g., clofibrate and gemfibrozil targeting PPARα and γ [57,58]. The pathogenic role of RORγT, which participates in the development of Th17 cells in the development of certain autoimmune diseases, has been recognized, and RORγT has become a potential target for therapy against these diseases. Thus, many researchers have focused on the search for substances with inverse agonist activity against RORγT [36]. Nevertheless, due to the ability of Th17 cells to increase the immune response, at least in some types of cancer [59], and their use in adoptive cell therapy [60,61,62], an increasing number of researchers have also recognized the therapeutic potential of RORγT agonists [35,37].

In this study, we screened a kinase inhibitor library using a validated reporter cell line [37] and identified AZ5104 (Figure 1) as a compound with RORγ agonist activity. AZ5104 was able to induce the transcriptional properties of RORγ (Figure 3), and induce the RORγ-dependent expression of the *G6PC* gene (Figure 4). Molecular docking experiments revealed that AZ5104 binds to the LBD of RORγ/RORγT with a significant docking score (Table 1, Figure 5). However, in Th17 cells, we were not able to reach similar concentrations of the compound due to high toxicity, and we observed different biological effects of AZ5104, including the inhibition of *RORγT* expression (Figure 6A) and RORγT-dependent transcription (Figure 6B–G). All these observations are consistent with the hypothesis that the observed effects of AZ5104 in Th17 cells are not mediated by binding of AZ5104 to the LBD of RORγT but rather by the ability of this compound to inhibit downstream elements of EGFR signaling. This is further supported by observations that AZ5104 inhibited pSRC, pERK1/2, and pSTAT3 in Th17 cells in a dose-dependent manner (Figure 8) but did not in HepG2 cells (Figure S7). STAT3 plays a crucial role in the development of Th17 cells [11,12], and its activity (through Tyr705 phosphorylation) is regulated by EGFR, SRC and ERK [63,64,65,66]. In that respect, our results are in line with previous observations that AhR-dependent activation of SRC and STAT3 is linked to enhanced Th17 differentiation [67] and that inhibition of ERK leads to inhibition of Th17 development [68]. To our knowledge, our report is the first showing the potential involvement of EGFR signaling in Th17 differentiation, adding another element to the complex process regulating the development of these lymphocytes. Epithelial growth factor (EGF)-mediated activation of EGFR has already been implicated in disorders with increased immunoreactivity, including asthma [69], in which Th17 cells may play an important pathogenic role [70,71]. Interestingly, cancer patients treated with EGFR inhibitors are more susceptible to *S. aureus* [72,73], a pathogen that is neutralized by Th17 lymphocytes [2]. In light of our results, this might be due to a decrease in the number of Th17 cells.

AZ5104, which was synthesized as an irreversible inhibitor of EGFR [40], is a demethylated metabolite of AZD9291 (osimertinib) [74,75]. The latter compound was approved by the Food and Drug Administration and the European Medicines Agency to treat T790M EGFR-positive non-small cell lung cancer [76]. The concentration of AZD9291 in patient serum can reach up to 795 nM [77], while another study [78] reported an average concentration of 409 ± 152 nM which is higher or very close to the concentrations of AZ5104 used in the present study (assuming that both compounds have a similar pharmacokinetic profile).

## 4. Materials and Methods

### 4.1. Cell Culture and Reagents

The HepG2 cell line (human hepatocellular carcinoma) was purchased from ATCC (Manassas, VA, USA) and maintained under standard conditions in Dulbecco’s Modified Eagle’s Medium (DMEM) (PAN Biotech GmbH, Aidenbach, Germany) supplemented with 10% fetal bovine serum (PAN Biotech GmbH) at 37 °C in an atmosphere of 5% CO2. The RORγ-HepG2 reporter cell line stably transfected with the reporter plasmid (RORE)6-tk-Luc [79] was described in our previous study [37] and was cultured in the same medium as the parental HepG2 cell line with the addition of 50 µg/mL hygromycin B (PAN Biotech GmbH). The HepG2-pGL4.35 reporter cell line was described in our previous study [80] and was maintained in DMEM (PAN Biotech GmbH) supplemented with 50 µg/mL hygromycin B (PAN Biotech GmbH). AZ5104 was purchased from Cayman Chemical (Ann Arbor, MI, USA).

### 4.2. Screening of the Chemical Library

The L1600 Kinase Inhibitor Library was purchased from TargetMol (Boston, MA, USA). RORγ-HepG2 cells were seeded into 96-well white plates. The next day, they were incubated with 1 µM concentration of compounds from the tested library for 24 h. Following incubation, the cells were harvested and lysed, and luciferase activity in the cell lysates was determined using an Infinite^®^ 200 PRO (Tecan) with d-Luciferin (luciferase substrate) (Cayman Chemical). A 2-fold induction of transcriptional activity was used as the cut-off value for the selection of positive compounds in an initial screening.

### 4.3. Cell Viability

The cytotoxicity of AZ5104 in HepG2 cells was evaluated with the neutral red uptake assay [81]. The absorbance of each sample was determined spectrophotometrically at 550 nm using a Sunrise microplate reader (Tecan, Männedorf, Switzerland). The cytotoxicity of AZ5104 in Th17 cells was determined using the CellTiter-Glo^®^ Luminescent Cell Viability Assay (Promega, Fitchburg, WI, USA) according to the manufacturer’s protocol. The luminescence of each sample was determined with an Infinite^®^ 200 PRO (Tecan, Männedorf, Switzerland).

### 4.4. Vectors, Transfection and Luciferase Assay

The RORγ expression plasmid was described previously [37]. Human RORγT, RORα, mouse Rorγ and Rorγt, and control pCMV-XL5 vectors were purchased from OriGene Technologies (Rockville, MD, USA). The RORβ expression vector was described previously [82] and was a kind gift from Roland Schϋle. The GAL4-DBD RORγ fusion construct was described previously [83] and was a kind gift from Patrick Griffin. RORγ-HepG2 cells were seeded into 96-well white plates. The next day, they were incubated with increasing concentrations of AZ5104 for 24 h. Following incubation, the cells were harvested and lysed, and luciferase activity in the cell lysates was determined using an Infinite^®^ 200 PRO (Tecan) with d-Luciferin (luciferase substrate) (Cayman Chemical). In experiments involving the expression vectors, the RORγ-HepG2 cells were cotransfected with the pCMV-SEAP vector (a kind gift from S. Schlatter, Zurich) as a control for transfection efficiency. Alkaline phosphatase activity in culture medium was determined spectrophotometrically at 405 nm.

### 4.5. Naive CD4+ T Cell Isolation and Differentiation into Th17 Cells

To obtain fully differentiated human Th17 lymphocytes, the protocol described previously by Wilson et al. [84] was used. Peripheral blood mononuclear cells (PBMCs) were isolated from buffy coats by centrifugation through Ficoll. Buffy coats from healthy, anonymous donors were bought as waste material from the Regional Center for Blood Donation and Blood Treatment (Łódź, Poland). The naive CD4+ fraction was isolated using CD4 M-pluriBeads^®^ anti-hu (pluriSelect Life Science, Leipzig, Germany). After isolation, cells were cultured for 5 days under Th17-polarizing conditions: Yssel’s medium containing human AB serum, cytokines (50 ng/mL human IL-1β, 30 ng/mL human IL-6, 10 ng/mL human IL-23, and 10 ng/mL human TGF-β) and beads coated with anti-CD2, anti-CD3, and anti-CD28 antibodies (T cell activation/expansion kit from Miltenyi Biotec, Bergisch Gladbach, Germany). The cytokines were purchased from PeproTech (Rocky Hill, NJ, USA).

### 4.6. Real-Time RT-PCR

TRI Reagent (Sigma Aldrich, St. Louis, MO, USA) was used to extract RNA, which was reverse transcribed using the Maxima First Strand cDNA Synthesis Kit for RT-quantitative PCR (Thermo Fisher Scientific, Waltham, MA, USA). Real-time RT-PCR was run on a LightCycler 480 from Roche (Basel, Switzerland) using SYBR Green I Master Mix. The reaction conditions were as follows: 95 °C for 5 min, followed by 40 cycles of 95 °C for 10 s, 60 °C for 10 s, and 72 °C for 20 s. We used the following primers: *G6PC*, 5’-TCCATACTGGTGGGTTTTGG-3’ (forward) and 5’-GAGGAAAATGAGCAGCAAGG-3’ (reverse) [37]; *RORγT*, 5′-CTGCTGAGAAGGACAGGGAG-3′ (forward); *RORγ*, 5′-CACAGAGACAGCACCGAGC-3′ (forward) and *RORγT/RORγ*, (same for both isoforms), 5′-AGTTCTGCTGACGGGTGC-3′ (reverse); *IL-17A*, 5′-AAACAACGATGACTCCTGGG-3′ (forward) and 5′-CTTGTCCTCAGAATTTGGGC-3′ (reverse) [28]; *IL-17F*, 5′-CTTTCTGAGTGAGGCGGC-3′ (forward) and 5′-TGGGAACGGAATTCATGG-3′ (reverse) [85]; *IL-9*, 5′-TCCCTCTGACAACTGCACC-3′ (forward) and 5′-GTGGTTTGGTTGCATGGC-3′ (reverse); *IL-21*, 5′-TCCCAAGGTCAAGATCGC-3′ (forward) and 5′-CCCTGCATTTGTGGAAGG-3′ (reverse) [79]; *IL22*, 5′-TGGCTGATAACAACACAGACG-3′ (forward) and 5′-GCTTTTGCACATTCCTCTGG-3′ (reverse); *IL23A*, 5′-GTTCCCCATATCCAGTGTGG-3′ (forward) and 5′-GACTGAGGCTTGGAATCTGC-3′ (reverse); *CSF2*, 5′-AGCCTCACCAAGCTCAAGG-3′ (forward) and 5′-AGTCAAAGGGGATGACAAGC-3′ (reverse); and *FOXP3*, 5′-AGGGCACAATGTCTCCTCC-3′ (forward) and 5′-GAGGAACTCTGGGAATGTGC-3′ (reverse). The mRNA levels were normalized by the geometric mean of the housekeeping genes *HPRT1*, *HMBS*, and *RPL13A,* as described by Vandensompele et al. [86].

### 4.7. siRNA Methodology

To downregulate the expression of the *RORC* gene, the duplexes of Stealth siRNA against RORC (HSS109302) transcript were used (Invitrogen, Carlsbad, CA, USA). As a negative control, siRNA-A with a scrambled sequence was used (Santa Cruz, Dallas, TX, USA). HepG2 cells were nucleofected with each siRNA using Amaxa SF Cell Line for 4DNucleofector™ X Kit L (Lonza, Basel, Switzerland) according to the manufacturer’s protocol.

### 4.8. Chromatin Immunoprecipitation (ChIP)

To perform ChIP, CD4+ cells were cultured in Th17 polarizing conditions in the presence of 0.4 µM AZ5104 for 5 days. The cells were fixed with formaldehyde to cross-link proteins with DNA, harvested, and lysed, and the DNA was subjected to sonication with a VCX-130 sonicator (Sonics & Materials Inc., Newtown, CT, USA). For chromatin immunoprecipitation, the EZ-Magna ChIP A/G Kit from EMD Millipore (Billerica, MA, USA) was applied. The following antibodies were used: normal mouse IgG (EMD Millipore) and anti-ROR gamma antibody [162C2a] (ab58670, Abcam, Cambridge, UK). The relative enrichment of the *IL17A* and *IL17F* promoters was analyzed with real-time PCR methodology using a SYBR Green I Master Mix on a LightCycler 96 from Roche. The reactions were run under the following conditions: 95 °C for 10 min, followed by 40 cycles of 95 °C for 20 s, 58 °C for 20 s, and 72 °C for 20 s. Primers complementary to *IL17A* and *IL17F* were described previously [80]: *IL17A*, 5′-GCAGCTCTGCTCAGCTTCTA-3′ (forward) and 5′-GGGCTTTTCTCCTTCTGTGG-3′ (reverse); and *IL17F*, 5′-CTCTGATTTGTGGGCAATGG-3′ (forward) and 5′-CCGGAGTTACTGACGAATGC-3′ (reverse). Soluble chromatin collected before immunoprecipitation was amplified as input control. To calculate the relative abundance of a specific promoter sequence enriched by protein-specific immunoprecipitation, we used the dCt method with the Ct obtained for input DNA as a reference value as follows: 1000 × 2 ^−dCt^, where dCt = Ct sample − Ct input.

### 4.9. Glucose 6-Phosphate Assay

To determine the amount of glucose-6-phosphate, the high sensitivity glucose 6-phosphate assay (Sigma Aldrich) was used according to the manufacturer’s instruction. HepG2 cells were treated with increasing concentrations of AZ5104 for 24 h. After that time, cells were harvested and homogenized. Cell lysates were deproteinized with an Amicon Ultra-0.5 Centrifugal Filter Unit (EMD Millipore) prior to addition to the reaction. Fluorescence intensity (excitation = 535 nm/ emission = 587 nm) was measured using Infinite^®^ 200 PRO (Tecan).

### 4.10. Detection and Quantification of IL-17 (ELISA)

Naive CD4+ cells were cultured under Th17 polarizing conditions in the presence of selected concentrations of AZ5104 for 5 days. Then, the culture supernatants were collected, and the concentration of IL-17 was analyzed by ELISA using the Quantikine Human IL-17 Immunoassay kit (R&D Systems). The absorbance of the samples at 405 nm was determined in a Sunrise microplate reader (Tecan).

### 4.11. Western Blotting

Whole cell lysates were prepared using RIPA buffer supplemented with a halt protease inhibitor cocktail (Thermo Fisher Scientific). Primary antibodies against the following proteins were used: beta-actin (ab8227, Abcam), ROR gamma antibody [162C2a] (ab58670, Abcam), G-6-Pase antibody (ab93857, Abcam), Src (2108, Cell Signaling, Danvers, MA, USA), phospho-Src family (2101, Tyr416, Cell Signaling), ERK 1/2 (sc-514302 C-9, Santa Cruz Biotechnology, Dallas, TX, USA), and phospho-ERK (sc-7383 E-4, Santa Cruz Biotechnology), STAT3 (9139, Cell Signaling), and phospho-Stat3 (4113, Tyr705, Cell Signaling). Specific bands were visualized using SuperSignal West Pico chemiluminescent substrate (Thermo Fisher Scientific) on the G-Box chemiluminescence imaging station (Syngene, Cambridge, UK). Quantifications of the bands were performed using ImageJ (http://imagej.nih.gov/ij/). Full scans of the original, uncut gels are available in the supplementary file.

### 4.12. Docking Simulations

All docking simulations were performed as previously described [37,80] with the following modifications: within each molecular docking simulation, the AZ5104 molecule was considered as a ligand. The initial molecular information was obtained from the Pubchem database [87] as a SMILES code. This molecular representation had been turned into the 3D geometry with the OpenEye Toolkit [88,89]. As host structures, the ligand binding domains of RORγ with the PDB IDs 3B0W [90] and 3L0J [91] were considered.

### 4.13. Statistics

Statistical analysis was performed using one-way ANOVA followed by Tukey’s post hoc test. The results obtained from human donors were analyzed using the Friedman repeated measures ANOVA on ranks followed by Tukey’s post hoc test. A *p*-value of 0.05 or lower was considered statistically significant.

## 5. Conclusions

AZ5104 and AZD9291 are potentially interesting structures in the context of developing novel treatments for autoimmune diseases, though one should remember that these compounds have a rather high rate of side effects [92,93,94] and thus, new, less toxic analogs are needed.

## Figures and Tables

**Table 1:** The lowest and mean free energy of binding for each considered host domain.

Host Domain	Estimated Free Energy of Binding (kcal/mol)		Number of Items in Cluster
	Lowest	Mean	
3L01	−9.53	−8.83	3
3B0W_A	−8.90	−8.90	1
3B0W_B	−9.04	−9.04	1

## Supplementary Materials

Supplementary materials can be found at https://www.mdpi.com/1422-0067/20/22/5780/s1.

## Abbreviations

ACT	adoptive cell therapy
AHR	aryl hydrocarbon receptor
BATF	basic leucine zipper ATF-like transcription factor
CD4+	CD4+ helper T cells
CSF2	colony stimulating factor 2
DBD	DNA-binding domain
EGF	epidermal growth factor
EGFR	epidermal growth factor receptor
ERK	extracellular regulated MAP kinase
FOXP3	forkhead box protein P3
G6PC	glucose-6-phosphatase catalytic subunit
GATA3	GATA binding protein 3
GM-CSF2	colony stimulating factor 2 (granulocyte-macrophage)
HMBS	hydroxymethylbilane synthase
HPRT1	hypoxanthine phosphoribosyltransferase 1
IL9	interleukin 9
IL17A	interleukin 17A
IL17F	interleukin 17F
IL-21	interleukin 21
IL-22	interleukin 22
IL-23	interleukin 23A
IRF4	interferon regulatory factor 4
LBD	ligand binding domain
NR1F3	nuclear receptor subfamily 1 group F member 3
PPAR	peroxisome proliferator-activated receptor
RORC	RAR-related orphan receptor gamma
RORγ	nuclear receptor ROR-gamma isoform 1
RORγT	nuclear receptor ROR-gamma isoform 2
RPL13A	ribosomal protein L13a
STAT3	signal transducer and activator of transcription 3
SRC	SRC proto-oncogene, non-receptor tyrosine kinase
t-bet	cell-specific T-box transcription factor
TGF-β	transforming growth factor beta
Th17	T-helper 17 cells
