# Supplementary material for: The Dichotomous Nature of AZ5104 (an EGFR Inhibitor) Towards RORγ and RORγT
Source: Int J Mol Sci. 2019 Nov 17;20(22):5780. doi: 10.3390/ijms20225780 (PMC6887705; doi:10.3390/ijms20225780)
Supplement: Supplementary file 1 [file ijms-20-05780-s001.zip › Supplementary information.doc]

Supporting information

The dichotomous nature of AZ 5104 (an EGFR inhibitor) towards RORγ and RORγT.

Kaja Karaś, Anna Sałkowska, Iwona Karwaciak, Aurelia Walczak-Drzewiecka, Jarosław Dastych, Rafał A. Bachorz, and Marcin Ratajewski

Table of contents

1. Supplementary Figure 1…………………………………………………………S3
2. Supplementary Figure 2…………………………………………………………S4
3. Supplementary Figure 3…………………………………………………………S5
4. Supplementary Figure 4…………………………………………………………S6
5. Supplementary Figure 5…………………………………………………………S7
6. Supplementary Figure 6…………………………………………………………S8
7. Supplementary Figure 7…………………………………………………………S9
8. Supplementary Table 1………………………………………………………….S10

Figure S1. Results of the screening of the L1600 Kinase Inhibitor Library using RORγ-HepG2 reporter cell line. 24 h after seeding cells were treated with compounds from the chemical library at a final concentration of 1 μM, and the cells were cultured for an additional 24 h. To determine transcriptional activity, the cells were lysed, and the cell lysates were analyzed for luciferase activity, as described in the Materials and Methods. Each point represents the average relative transcriptional activity from a single experiment. The line parallel to the x-axis represents a 2-fold induction of transcriptional activity, which was used as the cut-off value for the selection of positive compounds.

Figure S2. Cytotoxic effects of the AZ5104 in the fully differentiated Th17 cells determined with the Cell Titer-Glo assay. CD4+ naïve cells were isolated from buffy coats of the anonymous human donors and were differentiated under Th17-polarizing conditions for 5 days. After that time cells were treated with the increasing concentrations of the AZ5104 for 24 h. Mean ± SD, n = 6, *p < 0.05 compared with control treatment.

Figure S3. Effect of AZ5104 on the activity of human RORα and RORβ overexpressed in the RORγ-HepG2 reporter cell line. Mean ± SD, n = 5. Luciferase activity was standardized per corresponding secreted alkaline phosphatase activity, which was used as transfection efficiency control. Results were normalized to the activity of nontreated, pCMV-XL5-transfected cells.

Figure S4. Effect of AZ5104 and dowregulation of the human RORγ on expression of the G6PC gene in HepG2 cells. HepG2 cells were nucleofected with the control scrambled siRNA and siRNA against *RORC* and 24 h later were treated with AZ5104 (2μM) for another 24 h and then collected for RNA extraction. The mRNA expression of *RORγ* and *G6PC* was determined by real-time RT-PCR and normalized to that of the housekeeping genes *HPRT1*, *HMBS* and *RPL13A*. Mean ± SD, n = 4. *p < 0.05 compared with control treatment.

Figure S5. Effect of AZ5104 on IL-17 production that was determined using the Quantikine Human IL-17 Immunoassay kit (R&D Systems). Naive human CD4+ cells were treated with increasing concentrations of AZ5104 and cultured under Th17 polarizing conditions for 5 days. The data are presented as statistical dot plots with the median value (bars), n = 7.

Figure S6. Effect of AZ5104 on the expression of selected genes in fully differentiated human Th17 cells. Naive human CD4+ cells cultured under Th17 polarizing conditions for 5 days. Then, the cells were treated with the increasing concentrations of AZ5104 for 24h and after that time were collected for RNA extraction. The expression of the *RORγT* (A), *IL17A* (B), *IL17F* (C), *IL21* (D) genes was determined by real-time RT-PCR. The results were normalized to the housekeeping genes *HPRT1*, *HMBS* and *RPL13A*. *p < 0.05 compared with control cells. The data are presented as statistical dot plots with the median value (bars), n=6.

Figure S7. Effect of AZ5104 on the phosphorylation of kinases downstream of EGFR in HepG2 cells. HepG2 cells were treated with increasing concentrations of AZ5104 for 1 h and then lysed, and pSRC, pERK1/2 and pSTAT3 levels were analyzed using Western blotting (left panel). Results of densitometric analysis performed using ImageJ (right panel). Data are presented as mean ± SD, n = 3.

Table S1. Compounds positive in the first stage and after the second stage of screening for RORγ activators.

| Compound | Confirmed in reporter system | G6PC mRNA induction |
| --- | --- | --- |
| KenPaullone | yes | no |
| CUDC-101 | yes | no |
| BMS-265246 | yes | no |
| OTSSP167 | yes | no |
| Linifanib (ABT-869) | yes | no |
| AZD5438 | yes | no |
| WS6 | yes | no |
| AZ5104 | yes | yes |
| Bisindolylmaleimide IX Mesylate | yes | no |
| CVT-313 | yes | no |
| Chelerythrine chloride | yes | no |
| JNK-IN-7 | yes | no |
| MCB-613 | yes | no |
