# Supplementary material for: The Dichotomous Nature of AZ5104 (an EGFR Inhibitor) Towards RORγ and RORγT
Source: Int J Mol Sci. 2019 Nov 17;20(22):5780. doi: 10.3390/ijms20225780 (PMC6887705; doi:10.3390/ijms20225780)
Supplement: Supplementary file 1 [file ijms-20-05780-s001.zip › WesternBlots_IJoMS.doc]

Fig. 4

ROR gamma antibody [162C2a] ab58670

L-R control, 0.5μM AZ5104, 1μM AZ5104, 2μM AZ5104, marker

G-6-Pase antibody ab93857

L-R marker, control, 0.5μM AZ5104, 1μM AZ5104, 2μM AZ5104

beta-actin ab8227

L-R marker, control, 0.5μM AZ5104, 1μM AZ5104, 2μM AZ5104

Fig.8

Src

L-R marker, control, 0.1μM AZ5104, 0.2μM AZ5104, 0.4μM AZ5104

phospho-Src family Tyr416

L-R marker, control, 0.1μM AZ5104, 0.2μM AZ5104, 0.4μM AZ5104

ERK 1/2 C-9

L-R marker, control, 0.1μM AZ5104, 0.2μM AZ5104, 0.4μM AZ5104

phospho-ERK E-4

L-R marker, control, 0.1μM AZ5104, 0.2μM AZ5104, 0.4μM AZ5104

STAT3

L-R marker, control, 0.1μM AZ5104, 0.2μM AZ5104, 0.4μM AZ5104

phospho-Stat3 Tyr705

L-R marker, control, 0.1μM AZ5104, 0.2μM AZ5104, 0.4μM AZ5104

Beta actin

L-R marker, control, 0.1μM AZ5104, 0.2μM AZ5104, 0.4μM AZ5104

Supplementary Fig.7

Src

L-R marker, control, 0.5μM AZ5104, 1μM AZ5104, 2μM AZ5104

phospho-Src family Tyr416

L-R marker, control, 0.5μM AZ5104, 1μM AZ5104, 2μM AZ5104

ERK 1/2 C-9

L-R marker, control, 0.5μM AZ5104, 1μM AZ5104, 2μM AZ5104

phospho-ERK E-4

L-R marker, control, 0.5μM AZ5104, 1μM AZ5104, 2μM AZ5104

STAT3

L-R marker, control, 0.5μM AZ5104, 1μM AZ5104, 2μM AZ5104

phospho-Stat3 Tyr705

L-R marker, control, 0.5μM AZ5104, 1μM AZ5104, 2μM AZ5104

beta-actin ab8227

L-R marker, control, 0.5μM AZ5104, 1μM AZ5104, 2μM AZ5104
